# Strep A: challenges, opportunities, vaccine-based solutions, and economics

**DOI:** 10.1038/s41541-024-00863-7

**Published:** 2024-04-19

**Authors:** David E. Bloom, Jonathan Carapetis

**Affiliations:** 1 Harvard T.H. Chan School of Public Health, Boston, MA USA; 2 Wesfarmers Centre for Vaccines and Infectious Diseases, Telethon Kids Institute, University of Western Australia, Perth, WA Australia; 3 Perth Children’s Hospital, Perth, WA Australia

**Keywords:** Public health, Drug development, Vaccines

## Abstract

This collection of articles focuses on Streptococcus pyogenes (Strep A) vaccine research and innovation, with a focus on emerging efforts to understand and estimate the full societal value of Strep A vaccination.

*Streptococcus pyogenes* (also referred to herein as Strep A or S. pyogenes) is estimated to be the fifth-leading cause of infectious disease deaths globally, behind the human immunodeficiency virus (HIV), *Mycobacterium tuberculosis*, *Plasmodium falciparum*, and *Streptococcus pneumoniae* (although the advent of SARS-CoV-2 has likely pushed it to sixth place)^1^. Infections from Strep A occur across the entire age spectrum and result in perhaps the most diverse range of acute and chronic clinical manifestations of any known pathogen^2–4^. These include pharyngitis, impetigo, cellulitis, necrotizing fasciitis, streptococcal toxic shock syndrome, sepsis, maternal sepsis, scarlet fever, post-streptococcal glomerulonephritis, acute rheumatic fever (ARF), and rheumatic heart disease (RHD).

While Strep A infections are common globally, the burden of Strep A diseases varies widely according to country income level, socioeconomic and demographic composition of the local population, health system quality, and other factors. In high-income settings, for example, Strep A infection commonly manifests as pharyngitis (sore throat), which is typically treated with antibiotics quite effectively. Successful treatment of pharyngitis at this early stage can prevent rheumatic fever and may reduce the risk of invasive disease transmission to household contacts, although evidence that it can prevent other serious Strep A diseases is lacking^5,6^. Recent major upsurges in invasive Strep A infections have shown that penicillin alone will not control major outbreaks, and real-life experience in New Zealand shows that intensive antibiotic treatment of Strep A sore throat can reduce, at best, only about one-quarter of ARF episodes, at a cost that is unaffordable in low- and middle-income countries (LMICs)^7^. In addition, the proliferation of antibiotics used to treat pharyngitis presumed to be caused by Strep A introduces the real risk of antimicrobial resistance (AMR) both in the target Strep A organism but also in bystander pathogens where AMR is an even greater concern. Worse still, severe Strep A manifestations, such as RHD, disproportionately affect populations in low-resource settings and, consequently, perpetuate social inequities. During 2015–2017 in Australia, for example, the incidence rate of new first diagnosis of RHD among Indigenous people younger than 45 years of age was roughly 50 times higher than that of non-Indigenous people (45.5 vs. 0.9 cases per 100,000 person-years, respectively)^8^. Overall, the incidence of invasive Strep A infection has increased in several regions of the world during the past two decades^9–14^, and the health burden of Strep A is similar to, or higher than, that of other pathogens for which vaccines have recently been developed and successfully implemented (e.g., meningococcus).

Insofar as current approaches to prevention have proven insufficiently effective at the population level, combating Strep A infection and the diseases it causes requires a new approach. The Cannon and Wyber piece in this issue, “Modalities of group A streptococcal prevention and treatment and their economic justification,” explores various preventive interventions for Strep A infections and sequelae. The authors report that primordial prevention—behaviors like improved hygiene, physical distancing, and preventive vaccination—are particularly understudied, especially as averting infection prevents acute diseases like pharyngitis, impetigo, cellulitis, and necrotizing fasciitis and attendant downstream outcomes like RHD (and, therefore, also obviates the need for related downstream prevention and treatment strategies). Typically viewed as occurring passively with economic development, primordial prevention has long been neglected; indeed, of the 44 studies the authors reviewed that undertook economic analysis of Strep A prevention approaches, only one evaluated primordial prevention. One of the main conclusions of the Cannon and Wyber article is that a vaccine and related vaccination program could translate to a major advance in primary prevention—and potentially even primordial prevention—if it interrupts carriage and reduces the overall circulation of Strep A strains. As added gains, further interventions that address social determinants of health are likely to result in health and economic benefits for other infectious diseases, including educational and productivity gains.

Recognizing the persistently high global burden of Strep A infections, and considering the potential for prospective vaccines to address that health burden and its economic and social consequences, this special issue of *npj Vaccines* focuses heavily on investigating the value proposition for prospective Strep A vaccines. Entitled “Strep A: challenges, opportunities, and vaccine-based solutions,*”* this special issue provides a platform for diverse Strep A experts and stakeholders to inform the journal’s readers of ongoing research to advance Strep A vaccine development and implementation in an effort to widen and deepen our understanding of the full value of Strep A vaccines and vaccination. Included herein are original research, research protocols, reviews, and perspective pieces related to

- The global epidemiology and the health, social, and economic burden of Strep A
- A delineation of the myriad stakeholders who bear these costs
- A review of other modalities of Strep A prevention and treatment and their costs
- The history of Strep A vaccine development efforts and the current vaccine landscape
- Correlates of Strep A natural protection and immunity
- The case for Strep A vaccines, including the commercial value, cost-efficiency, and monetized global benefits of Strep A vaccines
- Opportunities for leveraging innovative frameworks and methodologies to comprehensively evaluate the full societal value of Strep A vaccination, and
- Estimates of optimal research and development (R&D) investments in the development of Strep A vaccines.

We view this special issue as a valuable opportunity to catalog contemporary challenges and opportunities in Strep A research in a respectable forum and to invite the journal’s readers to participate in advancing the Strep A research agenda. We also hope this special issue is seen as addressing a number of remaining knowledge gaps in Strep A vaccine research, and especially those gaps related to health technology assessments of Strep A vaccines and vaccination programs. We believe that prospective Strep A vaccines have been substantially undervalued, and an important dimension of the push for a vaccine is to remedy that deficiency. That undervaluation stems in part from health economists’ traditional reliance on methodologies for health technology assessment that adopt a narrow health payer–centric view of vaccination’s benefits. Such an approach neglects many health, economic, and social benefits, leading directly to underinvestment in vaccine development and coverage. This continued underinvestment is to both our individual and collective peril—especially in LMICs.

Reforming the health ecosystem to properly value and appropriately utilize vaccinations will ultimately require everything from reform of our institutions and practices for vaccine regulation; to advances in the way we conceptualize and operationalize health technology assessment; to increased investments in vaccine development, testing, and delivery. At its core, this means adopting a broad societal perspective, one that forces us to acknowledge squarely the fact that appropriately meaningful valuation of the health, economic, and social burdens of diseases is necessary for proper valuation of preventive, diagnostic, treatment, and surveillance interventions and for necessary systemic reforms^15,16^. Achieving the goals of this workstream requires that one be explicit and well aligned with respect to three sets of issues that are fundamental to undertaking meaningful assessments of value: perspective, sources of value, and metrics.

Perspective refers to the stakeholders whose interests we are using to assess value. For a comprehensive view of the picture, we must look at value assessment through the eyes of vaccine developers, manufacturers, and distributors; health ministers and finance ministers; vaccine recipients and their families; public and private donors; and society as a whole.

Sources of value refer to the dominant interests associated with each perspective. For vaccine manufacturers, value includes the risk-adjusted surplus of revenue above cost. For health ministers, it includes population health and healthcare spending. For finance and planning ministers, it includes public revenues and expenditures and the distribution of economic wellbeing. For individuals and their families, it includes the inherent and instrumental values of better health. For society as a whole, it includes the value associated with averted costs of Strep A disease, including the value assigned to any changes in social equity and political stability associated with reduced incidence and severity of vaccine-preventable diseases.

Metrics refer to how we measure value and set priorities among alternative potential uses of funds and need to have a clear and logical connection to the elements of perspective and sources of value. For vaccine manufacturers, a key metric is, of course, the commercial return on investment. For health ministers, a primary metric is incremental cost-effectiveness. For finance ministers, metrics include fiscal balance and macroeconomic performance. For society as a whole, a natural metric is a social benefit-cost ratio that reflects a broad range of health, economic, and social benefits attributable to spending on vaccine development and delivery.

Calculating a commercial rate of return, an incremental cost-effectiveness ratio, and a social benefit-cost ratio has well-established methods and data requirements, including due attention to the time frames over which different stakeholders bear costs and enjoy benefits. Each calculation answers a different question. Commercial rates of return are relevant for private investment decisions and are the bread and butter of corporate investment analysts. Cost-effectiveness ratios are relevant to allocating a health budget in the interests of maximizing population health and are the workhorse of standard health technology assessments. The field of health technology assessment has traditionally given short shrift to the full societal benefits of health interventions, but that problem is now being actively redressed, with a huge boost coming from what the COVID-19 pandemic has revealed about the health, economic, and social risks and burdens of infectious diseases and the corresponding benefits of preventive vaccines. Of course, there is a distinction to be made between vaccine development for pandemic preparedness and routine vaccination for protection against endemic diseases; the wholesale goals—who is paying, the return on investment, vaccine accessibility, vaccine efficacy, even acceptable side effects—vary in important ways between these different scenarios. Nevertheless, regardless of context, social benefit-cost ratios are relevant to maximizing societal welfare, which involves determining both the size of a health budget and its allocation among different programs and interventions.

An important guiding principle in this endeavor is that public health programs typically involve public costs. What needs greater appreciation is the fact that public costs are most naturally and appropriately compared with public benefits. First, in terms of the health benefits associated with vaccination, one issue that traditional, health-centric cost-effectiveness analyses commonly neglect is AMR. AMR is a dark, foreboding cloud hanging over humanity, and the value of vaccines in addressing that threat needs proper consideration and measurement. Vaccines can slow the development of AMR by preventing resistant infections and reducing the appropriate (and inappropriate) consumption of antibiotics. In terms of Strep A, resistance to penicillin, which is the first-line choice for treating superficial infections, has so far never been observed and is not a significant concern at present. However, Strep A resistance to other antibiotics used as treatments (e.g., macrolides) has been detected. Perhaps more worrisome, consumption of penicillin may engender beta-lactam resistance in bystander pathogens such as *Streptococcus pneumoniae*, where these antibiotics are the mainstays of treatment. Antibiotic consumption is especially high for both suspected and confirmed cases of Strep A pharyngitis. A recent study estimates that a Strep A vaccine administered globally to 5-year-olds could result in a 32% reduction in antibiotic prescriptions among 5−14-year-olds^17^. In addition to AMR, traditional cost-effectiveness analyses also neglect the value of preservation of the microbiome associated with reduced consumption of antibiotics. Microbiome disruption may lead to future infections or contribute to chronic ill health^18^. Cost-effectiveness analyses sometimes even neglect vaccination’s ability to interrupt disease transmission—the phenomenon at the heart of “herd protection.”

Routine health-centric cost-effectiveness analyses also typically fail to consider a slew of vaccination’s economic benefits. These benefits include better educational outcomes due to healthier kids having improved school attendance, higher educational attainment, and better cognitive function. These benefits are well documented for existing vaccines and are likely to contribute substantially to the full societal value of Strep A vaccines due to the disproportionate burden the pathogen places on children: the high incidence of pharyngitis and impetigo in kids (coupled with the transmissible nature of Strep A) leads to frequent absences among schoolchildren. Of course, research has consistently demonstrated increased educational attainment redounds to future employment and thus economic gains. Indeed, additional economic benefits include increases in labor force participation, hours worked, productivity, and adult earnings, including the additional income individuals generate when they are healthy enough to work themselves and are not required to care for ailing family members. A healthy populace also translates into an increased value of nonmarket time in various productive activities involving family and community, especially among older people. Notably, these labor-related economic impacts of Strep A vaccination are likely to be especially important in low-income settings where social safety nets are often lacking, familial care is common, and an individual’s labor is typically their greatest asset.

Relatedly, traditional health-centric cost-effectiveness analyses often omit important social benefits, such as improvements in social equity that result from the fact that vaccine benefits tend to accrue disproportionately to poor people who live in crowded conditions; vaccination buoys disadvantaged racial and ethnic minorities and vulnerable groups like women and children who may lack equitable access to healthcare—provided that the vaccine is widely available and not predicated on one’s ability to pay. Strep A vaccination may confer additional social benefits, such as a better quality of life—beyond improved health status—for individuals who would otherwise suffer the effects of long-term sequelae of Strep A diseases. Another social benefit of vaccination is the reduced stigma among RHD patients in populations where the disease is poorly understood.

Another article included here, “The full health, economic, and social benefits of prospective Strep A vaccination,” (by Cadarette et al.)^19^ employs a value-per-statistical-life-year (VSLY) approach to generate monetary estimates of prospective Strep A vaccination’s broad value. To avoid the undervaluation of benefits experienced by lower-income countries, which is somewhat inherent in the traditional VSLY method, the authors adopted a single global estimate of VSLY to be applied to all countries and assumed that it equals one to five times the global gross domestic product (GDP) per capita. Across several plausible vaccination scenarios, the VSLY-based value of reducing deaths and disabilities directly associated with Strep A vaccination in 30 birth cohorts is estimated to range from one to two trillion dollars if a safe and reasonably effective vaccine is administered at birth and from two to three trillion dollars if the vaccine is administered in early childhood. At the upper end, that sum is roughly equivalent to the United Kingdom’s pre-pandemic annual GDP.

Such striking amounts of potential benefit indicate that Strep A vaccination is likely to be a worthwhile investment and can aid determinations of how large of an investment into vaccine research and development stakeholders ought to make from the perspective of optimizing societal wellbeing. To that end, Dr. Daniel Tortorice led an effort aimed at building a rigorous model of optimal global R&D spending on Strep A vaccines, which includes a monetized estimate of the total harm caused by Strep A, the cost of R&D projects and the probability they will pass technical and regulatory hurdles, and the fraction of total harm an approved Strep A vaccine is expected to alleviate. The results point to optimal R&D spending that is considerably larger than actual spending. This important new line of inquiry underscores the fundamental point that undervaluation of health is perilous precisely because it leads to underinvestment in access and innovation (please see the piece in this issue entitled “Optimal global spending for group A *Streptococcus* vaccine research and development” by Tortorice et al. for further details)^20^.

And yet, many obstacles hinder the adoption of a full societal perspective in evaluating health interventions like Strep A vaccination. Among them is vaccine hesitancy, which can be at least partly addressed by building up trust—in science, in government, and in the health system—and through co-created initiatives with those with lived experience involving Strep A and its sequela.

Truly comprehending the scope of the vaccination landscape requires a larger aperture than has hitherto been used; that is, evaluating a health intervention necessitates incorporating all its components, from medical devices and pharmaceutical drugs to health system strengthening, in addition to non-health interventions such as transport, communication, energy, and schooling. Another issue is that the narrow health perspective that has dominated health technology assessments of vaccination inadequately reflects the time horizon for enjoying the benefits of avoiding childhood infectious disease, which tends to be much longer than for the prevention and treatment of disease among, for example, older people. It also does not reflect the fact that vaccination against infectious diseases tends to have substantially larger spillover and follow-on benefits than interventions against, for example, noncommunicable diseases. Finally, while high returns that may be revealed by a full societal analysis are necessary for an investment to be advisable, they may be insufficient to implement it; implementation requires funds that many cash-strapped countries simply do not have readily available. High returns do not guarantee affordability.

Therefore, funding high-value interventions like Strep A vaccination requires alternatives that would otherwise impose significant stress on public budgets. Philanthropic donations, such as funds from religious communities and from organizations like the Wellcome Trust, Open Philanthropy, the Leducq Foundation, and the Bill & Melinda Gates Foundation, are a promising source. Tax revenues, crowding out low-return or underperforming government programs, and the issuance of government debt (typically in the form of multiyear bonds) are other avenues of financing. This latter option—debt—is particularly appealing, as it allows close alignment of the time pattern of a vaccination program’s costs and benefits with the interest payments on a bond.

Financing is just one of many impediments hindering Strep A vaccine development. Safety concerns from adverse events in the 1960s led to a 30-year U.S. Food and Drug Administration (FDA)–imposed ban on Strep A vaccine testing in humans, setting the field back by decades. Therefore, key knowledge gaps remain, from scientific (such as the lack of vaccine-induced correlates of protection and how the diversity of the pathogen impacts the breadth of a vaccine candidate’s effectiveness), to developmental (the dearth of standardized assays to measure vaccine-induced immune responses), to regulatory (the target study design, population, and immunogenicity goals).

Momentum for a Strep A vaccine is finally building: the World Health Organization (WHO) declared it a priority in 2014, as did the Product Development for Vaccines Advisory Committee in 2016. WHO published an R&D roadmap for Strep A vaccine development in 2018. Then, the following year, the Strep A Vaccine Global Consortium (SAVAC) was established with support from the Wellcome Trust^21^. Through a network of specialists in epidemiology, health economics, medicine, immunology, and global health policy, SAVAC’s mission is to “ensure that safe, effective and affordable Strep A vaccines are available and implemented to decrease the burden of Strep A disease in the most in need” (https://savac.ivi.int). As a core part of SAVAC, those interrelated aspects of perspective, value, and metrics are being applied to prospective Strep A vaccines. In particular, SAVAC is developing a comprehensive, quantified Full Value of Vaccines Assessment of Strep A vaccines by conducting and considering analyses focused on the burdens of Strep A disease, the business investment case, the traditional health-centric (health payer–centric) investment case, and the global investment case.

Indeed, SAVAC is building a valuation framework for all stakeholders: commercial vaccine developers, health payers (such as a minister of health who must make decisions about the allocation of the health budget), and society-wide decision-makers (such as a minister of finance or a prime minister, who is obliged to consider the full health, economic, and social benefits of vaccination). That is, SAVAC is gathering the necessary data and parameters to determine whether Strep A vaccines are good spending opportunities, both privately and publicly—duly accounting for cross-community differences in epidemiological, social, economic, and health system contexts, and a host of uncertainties that take the form of sensitivity analyses. For the next phase of its endeavor, SAVAC 2.0 aims to push the Strep A vaccine initiative forward by collecting the key data points that are missing to help prepare candidates for vaccine trials, while engaging with industry and non-industry stakeholders (such as WHO, nonprofit funding organizations, policymakers, and technical advisors) to emphasize the mutual, multiple benefits of a Strep A vaccine and remove the obstacles that are hindering this effort.

Also established in 2019, the Australian Strep A Vaccine Initiative (ASAVI) was funded by the Australian government, with further contributions in 2021 by Open Philanthropy and the Leducq Foundation. ASAVI aims to accelerate the progression of a prospective Strep A vaccine to an efficacy trial for preventing pharyngitis^22^. Another research ally in this fight is STARFISH (STopping Acute Rheumatic Fever Infections to Strengthen Health), created in 2021 through a $5 million grant from Australia’s National Health and Medical Research Council. STARFISH has assembled a diverse team of researchers from Australia and the United States to fill critical knowledge gaps around environmental health and ARF. ARF and RHD disproportionately affect Aboriginal and Torres Strait Islander Australians. The STARFISH team is partnering with those communities to produce a comprehensive understanding of Strep A transmission and environmental health interventions that can reduce the risk of ARF.

These efforts are truly promising. Significant progress has been achieved in establishing the health burden of Strep A infection. Moving forward with a campaign to redouble investment efforts in Strep A vaccination requires establishing and connecting the economic and social burdens—and conversely the benefits that vaccination would confer—to that health burden. To convince stakeholders that significantly increasing investment into the R&D, manufacture, and delivery of Strep A vaccines is a worthwhile enterprise, more and better data must be collected. More comprehensive statistical models must be built from those data. The various sources of Strep A burden, beyond the morbidity and mortality directly attributable to the pathogen—such as its relationship to AMR and microbiome disruption—must be emphasized. The health, economic, and social spillovers from Strep A diseases to the family and community members of afflicted individuals must be quantified. So, too, the economic return on vaccine investment must be assessed—and highly publicized. In short, making the case for Strep A vaccination requires global buy-in from all sectors of society.

We asked some of the world’s foremost experts in Strep A research to contribute to this effort. The following is a snapshot of their work that appears in this issue.

Our opening article, “Global economic burden per episode for multiple diseases caused by group A *Streptococcus*,” by Jung-Seok Lee, Sol Kim, Jean-Louis Excler, Jerome H. Kim, and Vittal Mogasale^23^, seeks to supply the evidence of Strep A economic burden that has heretofore been lacking. The global health and economic burden of more than 20 Strep A infections—from pharyngitis and superficial skin infections to more invasive conditions like ARF and RHD—is disproportionately concentrated in LMICs and Indigenous populations. In fact, the prevalence of RHD in children 5–14 years old is highest in sub-Saharan Africa at 5.7 per 1000 children, followed by the Pacific and Indigenous populations of Australia and New Zealand (3.5 per 1000) and southcentral Asia (2.2 per 1000). RHD causes the greatest number of deaths from Strep A (319,400 in 2015 alone), and an additional 111 million cases of Strep A pyoderma and 616 million cases of Strep A pharyngitis occur annually. The authors considered direct medical costs, direct nonmedical costs, and indirect costs, and separately extrapolated and aggregated these figures to estimate the economic burden per episode by World Bank income group. They found that “the average economic burden per episode ranged from $22 to $392 for pharyngitis, $25 to $2,903 for impetigo, $47 to $2725 for cellulitis, $662 to $34,330 for invasive and toxin-mediated infections, $231 to $6332 for ARF, $449 to $11,717 for RHD, and $949 to $39,560 for severe RHD across income groups.” This enormous economic burden sounds a clarion call for vaccine development to ameliorate Strep A’s medical, and attendant financial, devastation.

Our next paper, “Modalities of group A streptococcal prevention and treatment and their economic justification” by Jeffrey W. Cannon and Rosemary Wyber^24^, reviews the literature on economic evaluations of different strategies for combating Strep A. The authors examine primordial, primary, secondary, and tertiary prevention studies, which largely concentrate on reducing the duration of illness or averting the issues caused by ARF and RHD among patients presenting with sore throat. Few extant studies have looked at reducing the burden of Strep A among the general population, let alone considered the ability to pay for and administer such approaches. In fact, the authors found no economic evaluations for primordial prevention that included environmental or social factors or hygienic behaviors, none that looked at tertiary prevention of invasive infection (such as adjuvant therapy), and no studies that considered all the strategies along the etiological pathway from infection to severe disease. Economic modeling can supply cost-benefit analysis to the current dearth of guidance for treatment and prevention best practices; however, the elements of these models are more readily accessible for imminent clinical therapies than for prolonged or large-scale public health strategies. The authors indicate that “validated costs and consequences for a more diverse range of Strep A interventions are needed to ensure that policies maximize patient outcomes under budget constraints.” This consideration should especially include enhanced evaluation of emerging strategies, particularly vaccination.

The following article is entitled “The *Streptococcus* pyogenes vaccine landscape” by Donald R. Walkinshaw, Meghan E. E. Wright, Anne E. Mullin, Jean-Louis Excler, Jerome H. Kim, and Andrew C. Steer^25^. Vaccine development and clinical studies for Strep A have been ongoing for more than 100 years, yet scientific, regulatory, and commercial barriers persist, and the vaccine pipeline remains fairly meager. However, recent developments—such as the 2018 World Health Assembly recommendation to prioritize a vaccine to combat RHD and the establishment of SAVAC on the heels of the WHO-produced vaccine development roadmap—have reenergized the initiative: eight candidates are currently on a product development track, including four M protein–based candidates and four candidates designed around non–M protein antigens. These candidates have demonstrated proof of concept in preclinical models, while one has demonstrated immunogenicity in a Phase I trial, and four others will soon enter clinical trials. SAVAC has connected global experts across multiple domains of expertise to fast-track vaccine development; the consortium has emphasized the favorable cost-benefit ratio and investment return of a potential vaccine while identifying the remaining knowledge gaps that need closing, such as the paucity of data from LMICs, the necessity of identifying correlates of protection, and the importance of publicizing Strep A burden and how a vaccine could ameliorate it.

Related to the previous article is another entitled “Update on the development of group A *Streptococcus* vaccines” by Sowmya Ajay Castro and Helge C. Dorfmueller^26^. In addition to milder conditions like tonsillitis and impetigo, group A *Streptococcus* causes more severe invasive diseases such as sepsis, streptococcal toxic shock, and necrotizing fasciitis. Left untreated, Strep A infections are also responsible for the serious immune-related sequelae of inflammatory glomerulonephritis, ARF, and RHD, estimated at 40 million cases worldwide. The momentum from the 2018 WHO Strep A vaccine development roadmap and the subsequent establishment of SAVAC has helped lead to new research partnerships and funding commitments for a Strep A vaccine, which now sees three candidates being tested or scheduled for Phase I clinical trials: (1) StreptAnova, which targets M proteins found on the surface of 30 Strep A serotypes; (2) StrepInCor, which is a 55–amino acid peptide from the C-terminal region of the M protein; and (3) a candidate that combines two M protein epitopes with an epitope from the streptococcal anti-neutrophil factor, Spy-CEP. Additionally, buoyed by the success of mRNA-based COVID-19 vaccines, several non–M protein–based vaccine candidates are in preclinical trials in rodent models. Support from CARB-X, Open Philanthropy, and the Wellcome Trust have funded several research groups to explore the group A carbohydrate (GAC) as a vaccine candidate, because it is present in every Strep A isolate and could target all serotypes. These numerous promising avenues of Strep A vaccine research demonstrate a renewed vigor for developing a Strep A vaccine, which seems more within reach than ever before.

Our next piece is entitled “Progress towards a glycoconjugate vaccine against group A *Streptococcus*” by Keira Burns, Helge C. Dorfmueller, Brendan W. Wren, Fatme Mawas, and Helen A. Shaw^27^. Although vaccine development against any pathogen is a complex process, the development of a Strep A vaccine has had a particularly challenging trajectory: WHO, in fact, notes its vaccine status as impeded. Due to the nature of the bacteria—its differing virulence and “the complicated, diverse global epidemiology of circulating Strep A serotypes,” among other challenges—scientists have struggled to target one specific protein that could serve as a universal vaccine candidate to protect against all serotypes causing Strep A’s multitudinous infections. Lancefield serotyping identifies Strep A by its type-specific surface-exposed carbohydrates that bind to specific antibodies; the presence of the conserved GAC on *S. pyogenes’* surface provides the “A” in Strep A’s common name. Due to its conservation, surface exposure, and antigenicity, glycoconjugates containing Strep A have become a crucial target in the mission to build a universal Strep A vaccine candidate. Employing double-hit glycoconjugates that incorporate species-specific carrier proteins, along with GAC, seems quite promising. The building elements of a successful vaccine candidate must be thoughtfully chosen, with a particular eye on its affordability for LMICs. Embracing novel technologies, such as bioconjugation and Generalized Modules for Membrane Antigens, are therefore especially imperative.

“Correlates of immunity to group A *Streptococcus*: a pathway to vaccine development,” by Hannah Frost, Jean-Louis Excler, Shiranee Sriskandan, and Alma Fulurija, is our next entry^28^. Understanding human immunoresponse to Strep A is fundamental for the development of successful vaccines to prevent the vast morbidity and mortality attributed to Strep A infections. While no vaccine yet exists, scientists can examine natural immunity to Strep A to identify immune correlates of protection (CoP) to inform future vaccine development. CoP define the immune response that an infection—or a vaccine—would need to trigger to protect a person from future infection, and CoP assays “could replace the need for clinical endpoints in vaccine efficacy trials, reducing the requirement for lengthy and costly studies with the disease as an endpoint.” CoP assays could help alleviate many complications of preventing and treating Strep A, such as the often remote and underserved populations who suffer from it. Such assays would be easily transferable among laboratories and countries and could allow for ongoing surveillance of immunity in target communities. The next step would be to optimize the assays to detect antibodies from saliva and finger blood samples. However, challenges remain. Unlike some viral vaccines, bacterial vaccines may necessarily employ multiple mechanisms to achieve immunity, meaning that no single functional assay can replicate the desired protection. Additionally, defining CoP in the absence of verifiable clinical protection from vaccine trials would be extremely difficult. Yet such vaccine trials are plausibly on the horizon and so too is the potential for CoP for Strep A.

The authors of “*Streptococcus* pyogenes vaccine candidates do not induce autoimmune responses in a RHD model,” Simone Reynolds, Rukshan Ahamed Mohamed Rafeek, Adam Hamlin, Ailin Lepletier, Manisha Pandey, Natkunam Ketheesan, and Michael F. Good^29^, developed a candidate vaccine to protect against multiple strains of *Streptococcus pyogenes* infections. The candidate vaccine contains two synthetic peptides (the M protein epitope, p*17, and the IL-8 degrading *S. pyogenes* cell envelope proteinase—SpyCEP—epitope, K4S2) derived from *S. pyogenes* proteins. Preclinical data show that these peptide vaccines, with specific formulation, “can produce a robust antibody response able to protect immunized animals against lethal challenge with multiple strains of S. pyogenes.” The purpose of this study, the first of its kind, was to evaluate whether the vaccine candidate antigens would induce autoimmune complications in their rat valvulitis model. While the rats in the positive control group that received *S. pyogenes* rM5 developed significant cardiac and neurological pathologies, there was no evidence of these pathologies in the PBS negative control group or, crucially, in the rats administered either P*17-DT or K4S2-DT. While the safety of an antigen derived from the M protein requires further investigation, this study provides preclinical evidence of the safety of vaccine candidates p*17 and K4S2 and offers a blueprint for assessing the safety of vaccine candidates in humans.

Our next work is entitled “Vaccine-induced, but not natural immunity, against the Streptococcal inhibitor of complement (SIC) protects against invasive disease” by Lionel K. K. Tan, Mark Reglinski, Daryl Teo, Nada Reza, Lucy E. M. Lamb, Vaitehi Nageshwaran, Claire E. Turner, Mats Wikstrom, Inga-Maria Frick, Lars Bjorck, and Shiranee Sriskandan^30^. Invasive disease caused by *Streptococcus pyogenes* has been increasing over the last 40 years and is currently associated with a mortality of approximately 20%. Strains expressing the M1 protein, encoded by *emm1*, are overrepresented among invasive isolates—accounting for more than 30% of cases of necrotizing fasciitis and streptococcal toxic shock syndrome—and this strain expresses the SIC, one of several virulence factors connected to *emm1*’s capacity to cause severe infection. While previous studies have suggested a role for SIC in invasive disease in vivo, expression of SIC has not been linked to *S. pyogenes’* invasiveness in the clinical setting. The authors aimed to measure SIC production by *S. pyogenes* in vitro and in vivo and then determine whether immunity to SIC can be protective. They found that “despite the prevalence of naturally occurring anti-SIC antibodies in humans, these antibodies do not confer opsonophagocytic protection against S. pyogenes. In contrast, vaccine-induced antibodies against full-length SIC do promote killing of S. pyogenes in a whole-blood assay and, furthermore, provide protection against experimental invasive streptococcal disease.” While the immunization of mice and rabbits with full-length SIC provided protection against systemic bacterial dissemination, unlike vaccine-induced immunity, natural human immunity to SIC is directed against only one domain of SIC, and, as the authors write, “this is insufficient to confer immunity.” Yet multicomponent vaccines have seen success against other bacterial infections, and the authors conclude that employing SIC in a multicomponent vaccine could reduce the burden of disease caused by *emm1 S. pyogenes*.

“Modeling the potential health impact of prospective Strep A vaccines” by Fiona Giannini, Jeffrey W. Cannon, Daniel Cadarette, David E. Bloom, Hannah C. Moore, Jonathan Carapetis, and Kaja Abbas^31^, develops a mathematical model based on the WHO’s 2018 Preferred Product Characteristics of a Strep A vaccine to estimate possible health effects. Strep A causes a broad spectrum of diseases; is particularly pernicious for young people and older adults, especially those in LMICs; and, beyond the enormous health burden, is responsible for a sizable economic burden at the individual, household, and societal levels. Accounting for the target age of vaccination, vaccine efficacy, duration of immunity, and vaccination coverage, the model (the first to be calibrated to predict a range of Strep A diseases for more than 200 countries) projected the health impact of Strep A vaccination at the global, regional, and national levels and by country income category. Among their findings for the six strategic scenarios they modeled, the authors estimated that, based on Strep A vaccine introduction between 2022 and 2034, “vaccination at birth for 30 vaccinated cohorts could avert 2.5 billion episodes of pharyngitis, 354 million episodes of impetigo, 1.4 million episodes of invasive disease, 24 million episodes of cellulitis, and 6 million cases of RHD globally.” Beyond their intrinsic value, these health estimates are useful data points for generating economic assessments, which will aid in quantifying the full societal value of a potential Strep A vaccine.

Transitioning to an economic lens, the authors (Jung-Seok Lee, Vittal Mogasale, Sol Kim, Jeffrey Cannon, Fiona Giannini, Kaja Abbas, Jean-Louis Excler, and Jerome H. Kim) of “The potential global cost-effectiveness of prospective Strep A vaccines and associated implementation efforts against various infections caused by *Streptococcus* pyogenes: a model-based analysis”^32^ aimed to estimate the global cost-effectiveness of a potential Strep A vaccine on different Strep A disease manifestations. Superficial infections often lead to more invasive Strep A diseases, so prevention is more expedient than treatment, particularly when considering the AMR concerns that accompany the (over)use of antibiotics. Additionally, Strep A infections place a disproportionate economic burden on LMICs: three low-resource nations—India, Pakistan, and the Democratic Republic of the Congo—account for 73% of global cases of RHD, which caused more than 300,000 deaths in 2015. The authors therefore constructed their vaccine impact model, which aligned with the WHO’s Preferred Product Characteristics for Strep A vaccines, to account for various income groups. They found that “Strep A vaccination would be cost-effective if the maximum cost per fully vaccinated person were properly set according to the income group considered: $8 to $308 for pharyngitis, $6 to $216 for RHD, $0.2 to $56 for invasive infections, $1 to $153 for impetigo, $0.1 to $28 for cellulitis, and $37 to $489 for all disease states combined, at the threshold of 1 x GDP per capita.” In high-income countries, vaccination would be particularly cost-effective for superficial infections such as pharyngitis and skin infections; in lower-income settings, vaccination would be more cost-effective to prevent RHD. Surveying the complete impact that vaccination would have on the five disease manifestations evaluated clearly shows that vaccination against Strep A would be a robustly cost-effective prevention strategy.

Turning to the R&D landscape, our next piece is entitled “A Strep A vaccine demand and return on investment forecast to inform industry R&D prioritization decisions” by Donald R. Walkinshaw, Meghan E. E. Wright, Marni Williams, Tanya M. F. Scarapicchia, Jean-Louis Excler, Ryan E. Wiley, and Anne E. Mullin^33^. Investment in Strep A vaccine research and development is disproportionately low relative to its global disease burden. RHD has the greatest morbidity and mortality of Strep A diseases: of an estimated 639,000 deaths per year attributed to Strep A, RHD accounts for 73%. Yet unlike other high-burden communicable diseases, such as HIV and tuberculosis, RHD-related deaths are expected to remain constant until 2040. Indeed, despite promising evidence of potential Strep A vaccine efficacy—the demonstration of acquired natural immunity, encouraging preclinical data in animal models, and proof-of-concept studies with human challenge models—only three vaccine candidates in development have even advanced to a Phase 1 clinical trial stage. Among many other obstacles hindering its development, safety concerns from adverse events in the 1960s led to a 30-year FDA-imposed ban on Strep A vaccine testing in humans, severely hindering vaccine progress. This paper introduces a novel Strep A vaccine demand and financial forecast model featuring estimates of potential global demand, revenue, and profits for a prospective Strep A vaccine; the authors also incorporate a net present value analysis of return on capital investments required to develop the vaccine, traditional demand and return on investment approaches, an examination of candidates in development, proxy vaccines, and interviews with relevant stakeholders. A positive net present value was calculated for various vaccine scenarios and populations, including a global rollout by a multinational pharmaceutical corporation and a staged rollout by a developing country vaccine manufacturer. The results suggest a viable commercial market for a Strep A vaccine indeed exists, potentially informing both industry investment and governmental prioritization of Strep A vaccine R&D.

Our penultimate piece is entitled “The full health, economic, and social benefits of prospective Strep A vaccination” by Daniel Cadarette, Maddalena Ferranna, Jeffrey W. Cannon, Kaja Abbas, Fiona Giannini, Leo Zucker, and David E. Bloom^19^. In addition to the reductions in morbidity, mortality, and future healthcare costs customarily demonstrated by economic evaluations of vaccination, much contemporary research has established vaccination’s wide range of health, economic, and social benefits. This paper evaluates the full societal benefits of widespread vaccination against Strep A, employing a willingness-to-pay approach via the VSLY model. VSLY assessments capture “the value of continuing to experience the joys of life itself for a longer period and the value of any changes in wellbeing attributes (e.g., income or medical costs) associated with the risk reductions.” VSLY, therefore, accounts for individual-level broad benefits of vaccination, like protection against dysbiosis—the gut imbalance that can result from overreliance on antibiotics to fight Strep A—and other health benefits, increased earnings, and improved quality of life. It also captures spillover effects, such as the fact that family members will benefit from an individual’s vaccination through reduced caregiving costs. The authors estimate aggregate lifetime benefits for 30 global birth cohorts would range from $1.7 to $5.1 trillion. Nevertheless, as of February 2023, only eight promising Strep A vaccine candidates were in development. Combined with the demonstrated scientific feasibility of a Strep A vaccine, the authors’ projections present a robust argument for substantial investment in the development of a Strep A vaccine that would confer large and widespread economic, health, and societal benefits.

We conclude this special issue with “Optimal global spending for group A *Streptococcus* vaccine research and development,” by Daniel Tortorice, Maddalena Ferranna, and David E. Bloom^20^, which aims to calculate the globally optimal level of investment for Strep A vaccine development and to quantify the resulting benefits and social rate of return. Since the FDA lifted the ban on human subject testing of Strep A vaccines in 2004, Strep A has become a promising target for vaccine development. However, the question of how much R&D funding should be allocated to this effort remains unanswered, while the bacterium continues to cause more than 600,000 deaths and 600 million cases of pharyngitis annually. The authors developed a model of optimal spending on R&D for vaccines and treatments, taking as inputs total harm from the disease, the probability an R&D project succeeds, the cost of a project, and the fraction of total harm a successful project would alleviate. They assumed the perspective of a supranational organization that can allocate funding for projects seeking to develop a Strep A vaccine and find an estimated optimal spending to be $33 billion as of 2020. This spending reaps 2020 $1.63 trillion in benefits—equal to 2% of annual global GDP—and a real return of 22.3% per year for 30 years, which includes 9–10% higher income annually via increased schooling. Demonstrably, the investment in a Strep A vaccine represents a high-return use of public spending and could create enormous benefits for comparatively little cost. Public policy can promote this vaccine development both through the direct funding of projects and by facilitating creative financial mechanisms, such as pooling private capital to allow investors to diversify their R&D investment.

## Data Availability

The article did not rely on any public, clinical, or third-party datasets.
